# Database of twitter influencers in cryptocurrency (2021–2023) with sentiments

**DOI:** 10.1186/s13104-023-06548-z

**Published:** 2024-10-11

**Authors:** Kia Jahanbin, Mohammed Ali Zare Chahooki

**Affiliations:** Department of Computer Engineering, Yazd University, Yazd, Iran

**Keywords:** Twitter, Influencers, Cryptocurrencies, Sentiment analysis, Price trend prediction

## Abstract

**Objectives:**

With the expansion of social networks such as Twitter, many experts share their opinions on various topics. The opinions of experts, who are also known as influencers, can be very influential. Combining these tweets and the historical prices of cryptocurrencies makes it possible to predict their price trends accurately. A Hybrid of RoBERTa deep neural network and BiGRU has been used for Sentiment Analysis (SA). Sentiments of tweets can be of great help to investors to understand the future behavior of the market and manage the stock portfolio. Unlike the tweets that are only extracted using the cryptocurrency name hashtag, the tweets of this dataset have specialized opinions and can determine the market trend.

**Data description:**

The dataset created in this research concerns the opinions of more than 52 influencers (persons or companies) regarding eight cryptocurrencies. This dataset was collected through the Apify Twitter API for eight months, from February 2021 to June 2023. This dataset contains five Excel files and tweets, compound score, importance coefficient of each tweet, sentiment polarity, and historical prices of four cryptocurrencies: Bitcoin, Ethereum, Binance, and other information. These tweets cover the opinions of 52 influencers on more than 300 cryptocurrencies, although most comments are related to Bitcoin, Ethereum, and Binance. For this reason, three Excel files containing the historical prices of polarity and compound sentiment related to Bitcoin, Ethereum, and Binance cryptocurrencies have been placed separately in the dataset. The polarity of sentiment in these Excel shows the maximum number of polarities by applying the importance coefficient, which determines the dominant polarity of sentiment related to a particular day for the cryptocurrency.

**Supplementary Information:**

The online version contains supplementary material available at 10.1186/s13104-023-06548-z.

## Objective

It has been shown in state-of-arts [1–3] that sentiment analysis effectively predicts the price trend of cryptocurrencies. In this research, the tweets of 52 influencers specializing in cryptocurrency were extracted using the API of the APIFY website from February 2021 to June 2023. Influencers’ tweets are essential because they contain expert opinions and can determine the market’s direction. To the best of the authors’ knowledge, this dataset is the first to include influencer tweets about more than 300 cryptocurrencies and sentiment polarity, unlike other datasets [4–6] that only cover one or two cryptocurrencies through hashtag mining. In addition to the polarity of tweets, this dataset includes the combination of polarities and importance factors for each tweet. After extracting and preprocessing the tweets, their polarity is determined by the combined model of RoBERTa and BiGRU and using the attention layer. In the dataset, another column called “new_coins” represents the name of the cryptocurrencies mentioned in the tweet. The primary purpose of this dataset is to provide specialized tweets for different cryptocurrencies. Kia et al. [7] have tested the quality of the previous version of this dataset on 40 cryptocurrencies. This dataset helps researchers analyze the opinions published on Twitter on the trends of a wide range of cryptocurrencies. The introduced data package includes four exclusive datasets for Bitcoin, Ethereum, Binance, and Dogecoin, which contain information such as historical prices, sentiment polarity, and sentiment compound. The polarity of sentiment for these four cryptocurrencies on any particular day is the maximum sum of different polarities by applying the importance coefficient. For example, suppose there are three positive comments with an importance factor of 0.2 and 2 negative comments with an importance factor of 0.4 in i-th for cryptocurrency j. In that case, we consider the polarity of the cryptocurrency to be negative.

## Data description

This dataset is published as a package and has five datasets. Two datasets include tweets of 52 influencers specializing in cryptocurrencies. In these tweets, there are expert opinions about more than 300 cryptocurrencies. The tweets were collected between February 05, 2021, and June 12, 2023, through the Twitter API of the Apify website. These tweets in three datasets related to Bitcoin, Ethereum, and Binance cryptocurrencies are limited from January 01, 2023, to June 12, 2023, due to extreme price fluctuations and changing political conditions. However, the data related to the period from February 2021 to June 2023 is available in two other datasets, including tweets. After extracting tweets, preprocessing operations were performed on the collected tweets, including stopwords removal, limitation, and equalizations. Then, general tweets, that is, tweets that do not contain information about any cryptocurrency, are removed. The cleaned data is injected into the combined neural network of RoBERTa, BiGRU, and the attention layer, and the polarity and sentiment compound is obtained. Then, the importance coefficient has been calculated for each tweet. Calculating the importance coefficient is essential because it assigns a different importance coefficient to tweets according to users’ attention. In assigning this coefficient, the number of followers of the user is not taken into account. So, the importance of the tweet from the user’s point of view is calculated by considering the parameters of the number of likes, retweets, and comments. This dataset package has been made available along with Python codes for preprocessing and sentiment analysis of tweets based on the information in Table 1.

**Table 1:** Overview of data files/data sets

Label	Name of data file/data set	File types (file extension)	Data repository and identifier (DOI or accession number)
Data set 1	dataset_52-person-from-2021-02-05_2023-06-12_21-34-17-266_with_sentiment	Excel (.csv)	Mendeley Data (10.17632/8fbdhh72gs.5)[8]
Data set 2	btc_selected_with_sentiment_2023_01_02_2023_06_12	Excel (.csv)	Mendeley Data (10.17632/8fbdhh72gs.5)[8]
Data set 3	bnb_selected_with_sentiment_2023_01_02_2023_06_12	Excel (.csv)	Mendeley Data (10.17632/8fbdhh72gs.5)[8]
Data set 4	eth_selected_with_sentiment_2023_01_02_2023_06_12	Excel (.csv)	Mendeley Data (10.17632/8fbdhh72gs.5)[8]
Python code	Preprocessing and sentiment analysis	Python(.ipynb, .docx)	Mendeley Data (10.17632/8fbdhh72gs.5)[8]

The information of Dataset 1 is: (1) created_at, (2) favorite_count, (3) full_text, (4) reply_count, (5)retweet_count, (6)clean_text (7)importance_coefficient, (8) importance_coefficient_normalized, (9)new_coin, (10) score ,and (11) sentiment_type. In the mentioned features, “created_at” is the time of publishing the tweet, “favorite_count” is the number of likes of the tweet, “full_text” is the full text of tweet, “reply_count” is the number of times the tweet has been replied, “retweet _count” is the number of retweets, “clean_text” is the text after preprocessing, “new _coin” The name of the coin is mentioned in the tweet, “score” shows the score of each of the positive, negative, and neutral polarities, and “compound” shows the degree of the sentiment of the text. Datasets 2, 3, and 4 also include the feature of the historical prices of the three cryptocurrencies, Bitcoin, Ethereum, and Binance, along with the features of changes, compound, and sentiment_type. The changes column shows the change (positive or negative) of the cryptocurrency price on the i-th day compared to the (i-1)-th day.

## Limitations

There are no limitations in the datasets, and the accounts used in the datasets to extract tweets are public.

## Electronic supplementary material

Below is the link to the electronic supplementary material.

Supplementary Material 1

Supplementary Material 2

Supplementary Material 3

Supplementary Material 4

Supplementary Material 5

## Data Availability

The data and codes described in this paper can be freely and openly accessed on data.mendeley (https://data.mendeley.com/datasets/8fbdhh72gs/5, doi: 10.17632/8fbdhh72gs). Please see Table 1 and references [8] for details and links to the data. How to cite this Dataset: jahanbin, kia; Zare Chahooki, Mohammad Ali; Rahmanian, Fereshte (2023), “Database of influencers’ tweets in cryptocurrency (2021–2023).”, Mendeley Data, V5, doi: 10.17632/8fbdhh72gs.5.
