# Supplementary material for: Database of twitter influencers in cryptocurrency (2021–2023) with sentiments
Source: BMC Res Notes. 2024 Oct 11;17:303. doi: 10.1186/s13104-023-06548-z (PMC11470647; doi:10.1186/s13104-023-06548-z)
Supplement: Supplementary file 4 — Supplementary Material 4 [file 13104_2023_6548_MOESM4_ESM.docx]

#Install packages

!pip install transformers

!pip install xformers

!pip install yahooquery

!pip install yfinance

!pip install yahoofinancials

!pip install tensorflow_addons

#Add packages

import pandas as pd

from numpy import NaN

import spacy

from transformers import pipeline

import re

import nltk

from textblob import TextBlob

from nltk.corpus import stopwords

from nltk.stem import WordNetLemmatizer

from sklearn.pipeline import make_pipeline

from sklearn.preprocessing import MinMaxScaler

from sklearn.metrics import confusion_matrix, ConfusionMatrixDisplay

import matplotlib.pyplot as plt

import numpy as np

from sklearn.metrics import f1_score, roc_auc_score

from sklearn.feature_selection import SelectKBest, f_regression

from sklearn.model_selection import train_test_split

from tensorflow.keras.layers import Input, LSTM, Attention, Dropout, GlobalAveragePooling1D, Dense

from tensorflow.keras.models import Model

from tensorflow.keras.callbacks import EarlyStopping, ModelCheckpoint

from tensorflow.keras.utils import plot_model

import tensorflow as tf

from transformers import TFRobertaModel

from nltk.sentiment.vader import SentimentIntensityAnalyzer

from nltk.sentiment.util import *

from textblob import TextBlob

from yahoofinancials import YahooFinancials

from google.colab import drive

#drive.mount('/content/drive')

nltk.download('stopwords')

nltk.download('wordnet')

nltk.download('punkt')

nltk.download('brown')

##Preprocessing

# Preprocessing function

stop_words = set(stopwords.words('english'))

lemmatizer = WordNetLemmatizer()

def preprocess_text(text):

if isinstance(text, str):

# remove punctuation and special characters

text = re.sub(r'[^\w\s]', '', text)

# convert to lowercase

text = text.lower()

# tokenize text

tokens = nltk.word_tokenize(text)

# remove stop words

tokens = [token for token in tokens if token not in stop_words]

# lemmatize text

tokens = [lemmatizer.lemmatize(token) for token in tokens]

# join tokens back into text

text = ' '.join(tokens)

return text

file_path = 'dataset_50-person-from-2021-02-05_2023-06-12_21-34-17-266.csv'

# Load the Excel file into a DataFrame

df = pd.read_csv(file_path,encoding='ISO-8859-1')

# Remove rows with "na" values

df = df.dropna(subset=['full_text'])

# Fill missing values in 'full_text' column with an empty string

df['full_text'] = df['full_text'].fillna('')

# to lower text

df['full_text'] = df['full_text'].str.lower()

# Preprocess the 'full_text' column

df['clean_text'] = df['full_text'].apply(preprocess_text)

# Filter the DataFrame to keep rows where "created_at" is greater than or equal to 2021-01-01

df = df[df['created_at'] >= '2021-01-01']

import datetime

# Convert the "created_at" column to datetime format

df['created_at'] = pd.to_datetime(df['created_at'])

# Extract the date part from the datetime and convert it to the desired format

df['created_at'] = df['created_at'].dt.strftime("%Y-%m-%d")

##add importance_coefficient per tweets

df['importance_coefficient'] = df['retweet_count'] + 2 * df['favorite_count'] + 0.5 * df['reply_count']

# Find the minimum and maximum values of the importance coefficient

min_value = df['importance_coefficient'].min()

max_value = df['importance_coefficient'].max()

# Normalize the importance coefficient

df['importance_coefficient_normalized'] = (df['importance_coefficient'] - min_value) / (max_value - min_value)

# Sort the DataFrame based on the "created_at" column in ascending order

df = df.sort_values('created_at', ascending=True)

# Print the sorted DataFrame

df.head()

## Model 1: Aspect based sentiment analysis (RoBERTa)

access_token = "Your_Token_in_hugging_face"

# Function to extract aspects and sentiments

def extract_aspects_sentiments(text):

# Load the spaCy English model for aspect extraction

nlp = spacy.load("en_core_web_sm")

# Extract aspects from the text

aspects = []

doc = nlp(text)

for token in doc:

if token.pos_ in ["NOUN", "PROPN"]:

aspects.append(token.text)

# Load the sentiment analysis model with your Hugging Face API token

sentiment_model = pipeline(

"sentiment-analysis",

model="mrm8488/distilroberta-finetuned-financial-news-sentiment-analysis",

tokenizer="mrm8488/distilroberta-finetuned-financial-news-sentiment-analysis",

use_auth_token=access_token

)

# Extract sentiment for the entire text

sentiment_result = sentiment_model(text)[0]

overall_sentiment = sentiment_result["label"]

overall_score = sentiment_result["score"]

# Extract sentiment for each aspect

aspect_sentiments = []

for aspect in aspects:

aspect_text = text.replace(aspect, "<aspect>")

aspect_sentiment_result = sentiment_model(aspect_text)[0]

aspect_sentiment = aspect_sentiment_result["label"]

aspect_score = aspect_sentiment_result["score"]

aspect_sentiments.append((aspect, aspect_sentiment, aspect_score))

return overall_sentiment, overall_score, aspect_sentiments

# Truncate the text to a maximum sequence length of 512 tokens

df1['truncated_text'] = df1['clean_text'].str[:512]

# Apply the extraction function to each row

df1[['overall_sentiment', 'overall_score', 'aspect_sentiments']] = df1['truncated_text'].apply(extract_aspects_sentiments).apply(pd.Series)

# Save the DataFrame back to CSV

df1.to_csv('c1_c50_total_processed.csv', index=False)

## Model 2: RoBERTa+BiGRU an attention layer sentiment analysis

from transformers import RobertaTokenizer, TFRobertaModel

# split data into input and target variables

X = df1['clean_text'].values

y = pd.get_dummies(df1['polarity']).values

# Calculate the maximum sequence length from the input data

max_length = max([len(x.split()) for x in X])

print(f"Max length: {max_length}")

# split data into training and validation sets

X_train, X_val, y_train, y_val = train_test_split(X, y, test_size=0.3, random_state=42)

# create Roberta tokenizer and encode inputs

tokenizer = RobertaTokenizer.from_pretrained('cardiffnlp/twitter-roberta-base')

train_encodings = tokenizer(X_train.tolist(), max_length=max_length, padding=True, truncation=True, return_tensors='np')

val_encodings = tokenizer(X_val.tolist(), max_length=max_length, padding=True, truncation=True, return_tensors='np')

# define early stopping and model checkpoint

early_stopping = tf.keras.callbacks.EarlyStopping(monitor='val_loss', patience=3, restore_best_weights=True)

model_checkpoint = tf.keras.callbacks.ModelCheckpoint('best_model_res_2015.h5', monitor='val_accuracy', mode='max', verbose=1, save_best_only=True)

# create TensorFlow datasets

train_dataset = tf.data.Dataset.from_tensor_slices((

{'input_sentence': train_encodings['input_ids'], 'input_mask': train_encodings['attention_mask']},

y_train

)).batch(32).prefetch(1)

val_dataset = tf.data.Dataset.from_tensor_slices((

{'input_sentence': val_encodings['input_ids'], 'input_mask': val_encodings['attention_mask']},

y_val

)).batch(32).prefetch(1)

# define input layer with correct name and shape

inputs = {

'input_sentence': tf.keras.layers.Input(shape=(max_length,), dtype=tf.int32, name='input_sentence'),

'input_mask': tf.keras.layers.Input(shape=(max_length,), dtype=tf.int32, name='input_mask')

}

# define model architecture

roberta_model = TFRobertaModel.from_pretrained('cardiffnlp/twitter-roberta-base')

roberta_embeddings = roberta_model({'input_ids': inputs['input_sentence'], 'attention_mask': inputs['input_mask']})[0]

roberta_embeddings = tf.keras.layers.Dropout(0.2)(roberta_embeddings)

gru_output = tf.keras.layers.Bidirectional(tf.keras.layers.GRU(128, return_sequences=False))(roberta_embeddings)

gru_output = tf.keras.layers.Dropout(0.2)(gru_output)

attention_output = tf.keras.layers.Attention()([gru_output, gru_output])

output = tf.keras.layers.Dense(3, activation='softmax')(attention_output)

model_ro_res_2015 = tf.keras.models.Model(inputs=inputs, outputs=output)

model_ro_res_2015.compile(optimizer=tf.keras.optimizers.Adam(learning_rate=2e-5), loss='categorical_crossentropy', metrics=['accuracy'])

# train model with early stopping and model checkpoint

history = model_ro_res_2015.fit(train_dataset, epochs=50, validation_data=val_dataset, callbacks=[early_stopping, model_checkpoint])

## Model 3: Vader Sentiment analysis

import nltk

nltk.download('vader_lexicon')

from nltk.sentiment.vader import SentimentIntensityAnalyzer

sid = SentimentIntensityAnalyzer()

df_selected =df.copy()

df_selected['scores'] = df_selected['full_text'].apply(lambda Description: sid.polarity_scores(Description))

df_selected.head()

df_selected = df_selected.loc[:, ['created_at', 'favorite_count', 'full_text', 'reply_count', 'retweet_count',

'user/screen_name', 'clean_text', 'importance_coefficient','importance_coefficient_normalized',

'new_coins', 'scores']]

df_selected = df_selected.rename(columns={'user/screen_name': 'user_id'})

df_selected['compound'] = df_selected['scores'].apply(lambda score_dict: score_dict['compound'])

df_selected['sentiment_type']=''

df_selected.loc[df_selected.compound>0,'sentiment_type']='POSITIVE'

df_selected.loc[df_selected.compound==0,'sentiment_type']='NEUTRAL'

df_selected.loc[df_selected.compound<0,'sentiment_type']='NEGATIVE'

## Get historical price

def get_historical_prices(tickers, start_date, end_date):

data = {}

for ticker in tickers:

yahoo_financials = YahooFinancials(ticker)

historical_data = yahoo_financials.get_historical_price_data(start_date, end_date, "daily")

data[ticker] = historical_data[ticker]['prices']

dfs = []

for ticker, prices in data.items():

df = pd.DataFrame(prices)

df = df.drop('date', axis=1).set_index('formatted_date')

df.columns = [f"{ticker}_close", f"{ticker}_high", f"{ticker}_low", f"{ticker}_open", f"{ticker}_volume", f"{ticker}_adjclose"]

df['formatted_date'] = pd.to_datetime(df.index) # Add formatted_date column

dfs.append(df)

merged_df = pd.concat(dfs, axis=1)

return merged_df

tickers = ['BTC-USD', 'ETH-USD', 'BNB-USD','XMR-USD','MATIC-USD','XRP-USD','DAI-USD','DOT-USD']

start_date = '2023-01-01'

end_date = '2023-06-12'

btc_df = get_historical_prices(tickers, start_date, end_date)

# Move formatted_date column to the first position

btc_df = btc_df[["formatted_date"] + [col for col in btc_df.columns if col != "formatted_date"]]

# Delete duplicate formatted_date columns

btc_df = btc_df.loc[:, ~btc_df.columns.duplicated()]

print(btc_df.head())

##Draw plot

import matplotlib.pyplot as plt

# Prepare the data

df_draw = btc_df[['formatted_date', 'XRP-USD_volume', 'ETH-USD_volume', 'BTC-USD_volume','XMR-USD_volume'

,'DAI-USD_volume','DOT-USD_volume']]

df_draw['formatted_date'] = pd.to_datetime(df_draw['formatted_date'], format='%Y-%m-%d %I-%p')

df_draw.set_index('formatted_date', inplace=True)

# Create the plot

plt.figure(figsize=(12, 6)) # Set the figure size to 12 inches wide and 6 inches high

plt.plot(df_draw.index, df_draw['XRP-USD_volume'], label='Ripple')

plt.plot(df_draw.index, df_draw['ETH-USD_volume'], label='ETH')

plt.plot(df_draw.index, df_draw['BTC-USD_volume'], label='BTC')

plt.plot(df_draw.index, df_draw['XMR-USD_volume'], label='Monero')

plt.plot(df_draw.index, df_draw['DOT-USD_volume'], label='Polkadot')

#plt.plot(df_draw.index, df_draw['DAI-USD_volume'], label='Dai')

plt.xlabel('Date')

plt.ylabel('Volume')

plt.title('Cryptocurrency Volume Over Time')

plt.legend()

plt.show()

## Select BTC,BNB,ETH Data from CSV and add price and sentiment

btc_selected = btc_df.iloc[:, :7]

btc_selected = btc_selected.round(0).astype(int)

btc_selected['formatted_date'] = btc_selected.index

btc_selected.head()

#---ETH-selecte------------

eth_selected = btc_df.iloc[:, 7:13]

eth_selected = eth_selected.round(0).astype(int)

eth_selected['formatted_date'] = eth_selected.index

eth_selected

#--BNB-selected------------

bnb_selected = btc_df.iloc[:, 13:19]

bnb_selected = bnb_selected.round(0).astype(int)

bnb_selected['formatted_date'] = bnb_selected.index

# Calculate price changes

btc_selected['price_changes'] = btc_selected['BTC-USD_close'].diff()

btc_selected['price_changes'] = btc_selected['price_changes'].apply(lambda x: 'positive' if x > 0 else 'negative' if x < 0 else 'neutral')

# Calculate price changes

eth_selected['price_changes'] = eth_selected['ETH-USD_close'].diff()

eth_selected['price_changes'] = eth_selected['price_changes'].apply(lambda x: 'positive' if x > 0 else 'negative' if x < 0 else 'neutral')

eth_selected

# Calculate price changes

bnb_selected['price_changes'] = bnb_selected['BNB-USD_close'].diff()

bnb_selected['price_changes'] = bnb_selected['price_changes'].apply(lambda x: 'positive' if x > 0 else 'negative' if x < 0 else 'neutral')

bnb_selected

#change columns name

def remove_chars_before_underscore(df):

df.columns = df.columns.str.split('_').str[-1]

remove_chars_before_underscore(btc_selected)

remove_chars_before_underscore(eth_selected)

remove_chars_before_underscore(bnb_selected)

btc_selected

eth_selected

bnb_selected

## add sentimeni type and compund to dataframe

bnb_selected['date'] = pd.to_datetime(bnb_selected['date'])

bnb_sentiment['created_at'] = pd.to_datetime(bnb_sentiment['created_at'])

# Perform left merge on 'date' and 'created_at' columns

bnb_selected = pd.merge(bnb_selected, bnb_sentiment[['created_at', 'compound', 'sentiment_type']],

left_on='date', right_on='created_at', how='left')

# Drop the redundant 'created_at' column

bnb_selected = bnb_selected.drop('created_at', axis=1)

bnb_selected

## add sentimeni type and compund to dataframe

eth_selected['date'] = pd.to_datetime(eth_selected['date'])

eth_sentiment['created_at'] = pd.to_datetime(eth_sentiment['created_at'])

# Perform left merge on 'date' and 'created_at' columns

eth_selected = pd.merge(eth_selected, eth_sentiment[['created_at', 'compound', 'sentiment_type']],

left_on='date', right_on='created_at', how='left')

# Drop the redundant 'created_at' column

eth_selected = eth_selected.drop('created_at', axis=1)

eth_selected

## add sentimeni type and compund to dataframe

btc_selected['date'] = pd.to_datetime(btc_selected['date'])

btc_sentiment['created_at'] = pd.to_datetime(btc_sentiment['created_at'])

# Perform left merge on 'date' and 'created_at' columns

btc_selected = pd.merge(btc_selected, btc_sentiment[['created_at', 'compound', 'sentiment_type']],

left_on='date', right_on='created_at', how='left')

# Drop the redundant 'created_at' column

btc_selected = btc_selected.drop('created_at', axis=1)

btc_selected

# Drop the top row

btc_selected = btc_selected.iloc[1:]

# Output the merged dataframe

btc_selected

# Drop the top row

eth_selected = eth_selected.iloc[1:]

# Output the merged dataframe

eth_selected

# Drop the top row

bnb_selected = bnb_selected.iloc[1:]

# Output the merged dataframe

bnb_selected

## Save data in Excel

eth_selected.to_csv('eth_selected_with_sentiment_2023_01_02_2023_06_12.csv')

btc_selected.to_csv('btc_selected_with_sentiment_2023_01_02_2023_06_12.csv')

bnb_selected.to_csv('bnb_selected_with_sentiment_2023_01_02_2023_06_12.csv')
